# Dietary factors and risk for amyotrophic lateral sclerosis: A two sample mendelian randomization study

**DOI:** 10.1097/MD.0000000000038473

**Published:** 2024-06-21

**Authors:** Zhaoyi Jing, Xianghua Qi, Jing Teng

**Affiliations:** a Shandong University of Traditional Chinese Medicine, Jinan, China; b Shandong University of Traditional Chinese Medicine Affiliated Hospital, Jinan, China.

**Keywords:** amyotrophic lateral sclerosis, dietary intake, Mendelian randomization, nutritional adaptation

## Abstract

Correlations between dietary factors and amyotrophic lateral sclerosis (ALS) have been found in previous observational studies. However, no further studies have used Mendelian randomization to further explore the causal relationship between dietary factors and ALS. Clarifying these relationships is a crucial part of developing nutritional recommendations for ALS prevention. The exposure and outcome datasets employed in this study were extracted from the IEU Open GWAS project (https://gwas.mrcieu.ac.uk/). The exposure datasets involved in our Mendelian analyses consisted of meat intake (processed meat intake, poultry intake, beef intake, pork intake, non-oily fish intake, and oily fish intake), staple foods intake (bread intake and cereal intake), vegetable intake (cooked vegetable intake, salad/raw vegetable intake), fruit intake (fresh fruit intake and dried fruit intake), and beverage intake (coffee intake and tea intake). The weighted median, MR-Egger, Inverse Variance Weighted, Simple mode and Weighted mode methods were all utilized. And we applied Inverse Variance Weighted method as the main judgement criterion for Mendelian randomization analysis. Heterogeneity and pleiotropy analyses were conducted to confirm the validity of the outcomes. Genetically predicted that oily fish intake (OR: 0.7648; 95% CI: 0.5905–0.9904; *P* = .0420), coffee intake (OR: 0.7385; 95% CI: 0.5660–0.9637; *P* = .0256), and fresh fruit intake (OR: 0.6165; 95% CI: 0.4007–0.9487; *P* = .0278) were causally associated with a decreased risk of ALS. Negative results (*P* > .05) were received for all other dietary factors. This study found that oily fish intake, coffee intake and fresh fruit intake reduced the risk of developing ALS. Additionally, other factors were not associated with ALS.

## 1. Introduction

Amyotrophic lateral sclerosis (ALS) is the most common motor neuron disease with muscle weakness as the first symptom. It is characterized by degenerative changes in the upper and lower motor neurons.^[1,2]^ Patients typically experience respiratory failure and succumb to the disease within a span of 3 to 5 years from the onset of symptoms.^[3–5]^ The etiology of ALS is intricate and largely unknown, involving various factors such as age, genetics, environment,^[6]^ and potentially military deployment.^[7]^ The significance of these findings is paramount as numerous studies have explored ALS risk factors. However, the sole FDA-approved drug for ALS patients, Riluzole, only offers a modest extension of life by a few months.^[8]^ In the early stages of ALS, targeted adjustments to lifestyle and diet have demonstrated beneficial effects.^[9,10]^

The vital contribution of dietary factors in the course of ALS progression has been documented, according to a previous investigation.^[11]^ Dietary factors may be related to the onset of ALS.^[12,13]^ Former investigations have observed that fruit intake,^[14,15]^ vegetable intake,^[16]^ staple food intake,^[11,17]^ beverage intake,^[18]^ and meat intake^[19]^ were in relation to ALS. Mendelian randomization (MR), which uses genetic variation as an instrumental variable (IVs), is capable of effectively eliminating the interference of confounding factors and has certain advantages over other research methods.^[20]^ Nonetheless, there is a scarcity of MR studies investigating the causal links between dietary factors and ALS. In light of this, we conducted a comprehensive MR analysis to investigate the associations between dietary factors and ALS.

## 2. Methods

**Figure 1.**
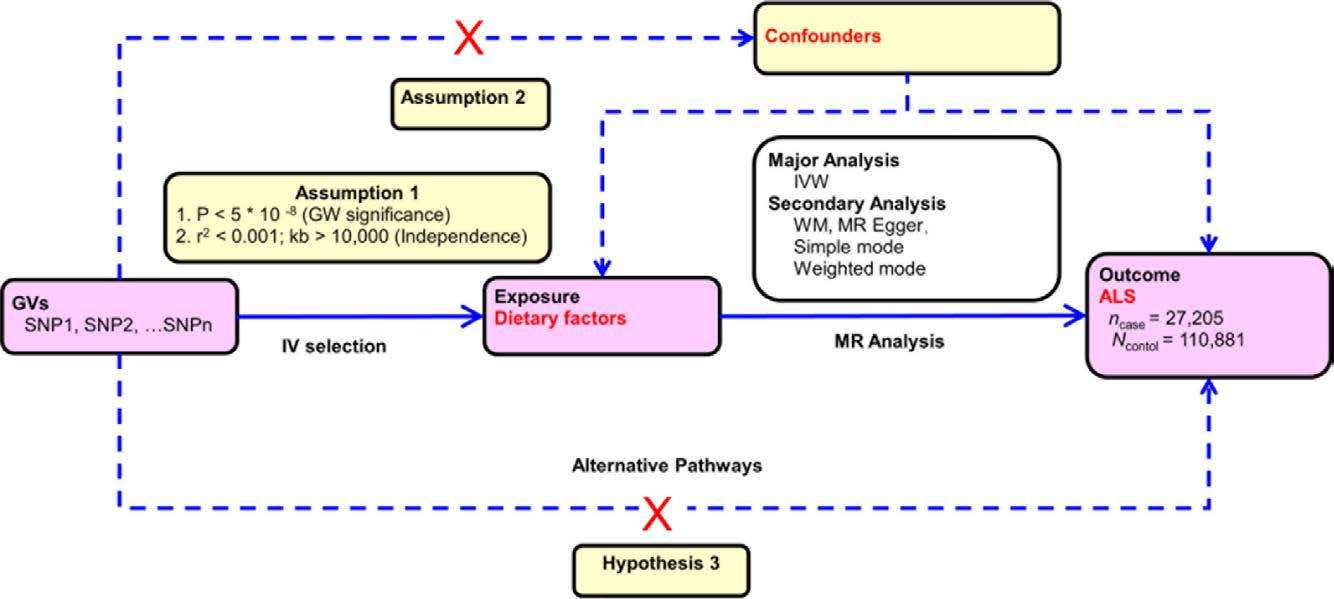
The overall design of Mendelian randomization analysis in the present study. Assumption 1, the genetic variants are supposed to be strongly associated with the risk of interest. Assumption 2, the genetic variants should not be associated with any confounding factors. Hypothesis 3, the genetic variants should affect the risk of the outcome only mediated by the exposures.

The premise of MR analysis consists of the following foundational assumptions, as illustrated in Figure 1. Firstly, the exposure factor(s) must exhibit a strong association with the IVs (Assumption 1). Secondly, the IVs should not be correlated with any potential confounding factors (Assumption 2). Thirdly, the IVs should not display direct correlation with the outcome variable (Hypothesis 3). To conduct this study, we utilized Genome-Wide Association Study (GWAS) summary-level data retrieved from the Integrative Epidemiology Unit (IEU) open GWAS project. The project, supported by the MRC IEU at the University of Bristol, compiled and analyzed GWAS data from multiple sources, including UK Biobank, published articles, and the European Bioinformatics Institute. It is noteworthy that ethical approval was not a requirement for this research since the data employed in this research was publicly available, anonymized, and de-identified.

### 2.1. Data sources

Factors related to dietary habits adopted in this research involved vegetable intake (salad/raw vegetable intake and cooked vegetable intake), meat intake (processed meat intake, poultry intake, beef intake, non-oily fish intake, oily fish intake, and pork intake), staple food intake (bread intake and cereal intake), beverage intake (coffee intake and tea intake), and fruit intake (dried fruit intake and fresh fruit intake). We extracted the GWAS summary-level data for dietary habits from the UK Biobank database via The IEU open GWAS project. And the GWAS summary-level data for ALS was obtained from the European Bioinformatics Institute by the IEU open GWAS project. To ascertain the single-nucleotide polymorphisms (SNPs) associated with the outcome, we refrained from utilizing proxy SNPs. This decision was primarily driven by the substantial number of SNPs available in the European Bioinformatics Institute dataset for ALS, amounting to 10,427,126 SNPs. Table 1 provides additional details on the exposure and outcome datasets.

**Table 1:** Information of the exposures and outcome datasets.

IEU GWAS ID	Exposure or outcome	Identified SNPs	Participants included in analysis
ukb-b-6324	Processed meat intake	23	461981 European-descent individuals
ukb-b-8006	Poultry intake	7	461900 European-descent individuals
ukb-b-2862	Beef intake	15	461053 European-descent individuals
ukb-b-17627	Non-oily fish intake	11	460880 European-descent individuals
ukb-b-2209	Oily fish intake	57	460443 European-descent individuals
ukb-b-5640	Pork intake	14	460162 European-descent individuals
ukb-b-11348	Bread intake	24	452236 European-descent individuals
ukb-b-15926	Cereal intake	37	441640 European-descent individuals
ukb-b-1996	Salad/raw vegetable intake	20	435435 European-descent individuals
ukb-b-8089	Cooked vegetable intake	10	448651 European-descent individuals
ukb-b-5237	Coffee intake	36	428860 European-descent individuals
ukb-b-6066	Tea intake	41	447485 European-descent individuals
ukb-b-16576	Dried fruit intake	37	421764 European-descent individuals
ukb-b-3881	Fresh fruit intake	50	446462 European-descent individuals
ebi-a-GCST90027164	Amyotrophic lateral sclerosis	NA	27205 European-descent cases and 110881 European-descent controls

GWAS = Genome-Wide Association Studies, IEU = Integrative Epidemiology Unit, NA = not applicable, SNPs = single-nucleotide polymorphisms.

### 2.2. The selection of IVs

MR analyses rely on IVs to investigate causal relationships between exposures and outcomes. This approach allows researchers to examine the effects of exposures on outcomes without the confounding effects of reverse causation or unmeasured variables. Using IVs in MR analyses can provide valuable insights into the causal relationships between exposures and outcomes. By leveraging genetic variations as proxies for exposures, researchers can explore potential causal pathways and mechanisms underlying complex diseases and health outcomes. The SNPs specifically associated with dietary factors were obtained from the IEU open GWAS project (https://gwas.mrcieu.ac.uk/). In the course of this analysis, we conducted a rigorous screening process to identify the SNPs that were highly relevant to exposures. SNPs were only considered if they reached genome-wide significance level (*P* < 5 × 10^–8^), had a clumping window larger than 10,000 kb, and exhibited a low level of linkage disequilibrium (*r*^2^ < 0.001). To guarantee a robust relationship between the IVs and exposure, we utilized the F statistic. It is widely recognized that a F statistic exceeding 10 satisfies the requirements for a strong association.^[21]^ Additionally, we performed searches through the PhenoScanner website (http://www.phenoscanner.medschl.cam.ac.uk/), which led to the startling removal of SNPs associated with confounders and outcome.^[22]^

### 2.3. Statistical analysis

The primary method used for calculating the causal effect in our study was the inverse variance weighted (IVW) method. The IVW model is known for its robustness in detecting causation in two sample mendelian randomization analysis. This method was chosen due to its superior ability to assess the causal relationship between variables.^[23]^ To ensure the robustness of the IVW results, additional sensitivity analyses were performed, including the weighted median, MR-Egger, simple mode, and weighted mode methods. The weighted median method enables satisfactory estimates when at least 50% of the IVs are valid instruments.^[24]^ We also implemented the “leave-one-out” (LOO) method to assess the influence of an individual SNP on the overall causal findings by excluding each genetic variant in turn and recomputing the MR–IVW estimates.

Heterogeneity in the IVW model was evaluated using Cochran’s Q test, which demonstrated significant heterogeneity when *P* < .05. However, the presence of heterogeneity does not necessarily mean that the IVW model is invalid. To verify the credibility of the IVs, we tested for horizontal pleiotropy using egger’s intercept and the MR-PRESSO method. For the MR–PRESSO method,^[25]^ The “global test” was utilized to examine the overall horizontal pleiotropy; The “MR-PRESSO outlier test” was applied to remove possible outliers in IVs and produce corrected results; and To evaluate the presence of distortion in the outcomes before and after correction, the “MR-PRESSO distortion Test” was performed. Similarly, we computed the egger intercept as an additional approach to investigate horizontal pleiotropy. When there was no obvious distance from the intercept to the origin, it was regarded as having no influence on pleiotropy.

All analyses were performed in R software version 4.3.2 by the “TwoSampleMR” package (version 0.5.6)^[26]^ and the “MR–PRESSO” package (version 1.0).^[25]^

## 3. Results

This research systematically analyzed the causal relationship between 14 different dietary habit factors and ALS. Following the removal of outliers, the number of SNPs extracted from the database ranged from 7 to 57 (with *F* statistics > 10). To guarantee the precision of the findings, the research specifically concentrated on individuals of European heritage. The exposure datasets were sourced from UK Biobank and the number of participants ranged from 421,764 to 461,981. The outcome dataset comprised 27,205 ALS cases of European descent and 110,881 controls from the European Bioinformatics Institute. Due to the utilization of GWAS data from 2 different genetic consortiums, there was minimal overlap between the populations involved in the exposures and outcome.

**Figure 2.**
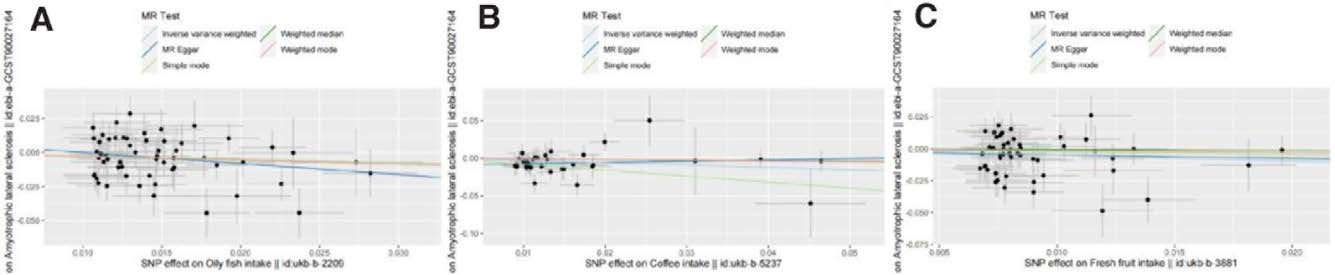
Scatter plots of MR analysis. The slope of each line corresponding to the estimated MR effect based on various models. (A) Oil fish intake, (B) Coffee intake, (C) Fresh fruit intake. MR = Mendelian randomization.

**Figure 3.**
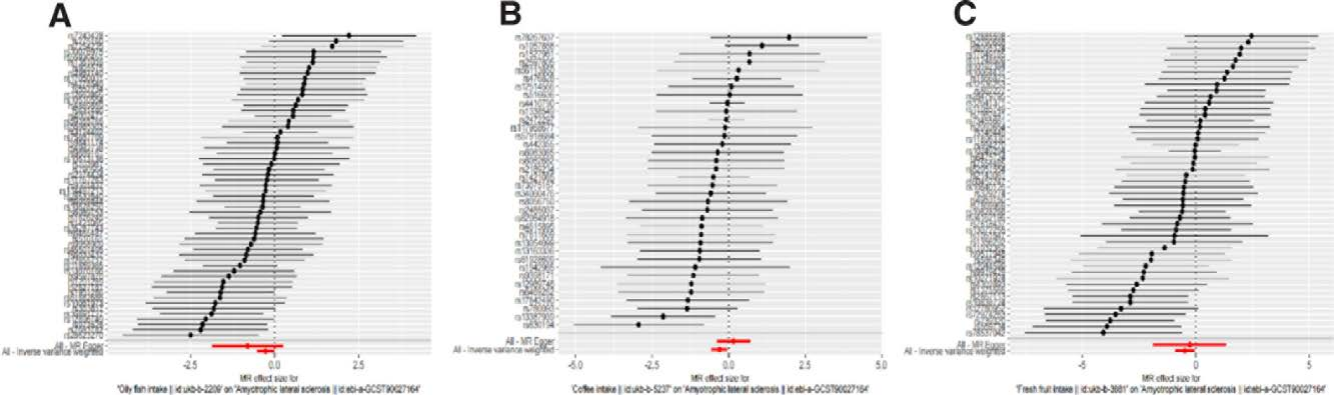
Forest plots of the causal effect of (A) Oil fish intake, (B) Coffee intake, (C) Fresh fruit intake on ALS. ALS = amyotrophic lateral sclerosis.

In this research, a total of 3 causalities were identified (*P* < .05 by IVW method). We obtained three positive results, including Oily fish intake (after eliminating outliers OR: 0.7648; 95% CI: 0.5905–0.9904; *P* = .0420), Coffee intake (OR: 0.7385; 95% CI: 0.5660–0.9637; *P* = .0256, no outliers), and Fresh fruit intake (after eliminating outliers OR: 0.6165; 95% CI: 0.4007–0.9487; *P* = .0278). The scatter plots and the forest plots corresponding to the exposure factors of these three positive results are shown in Figures 2 and 3. Based on the odds ratios and 95% confidence intervals, these three dietary habits were proved to be protective factors against ALS.

**Figure 4.**
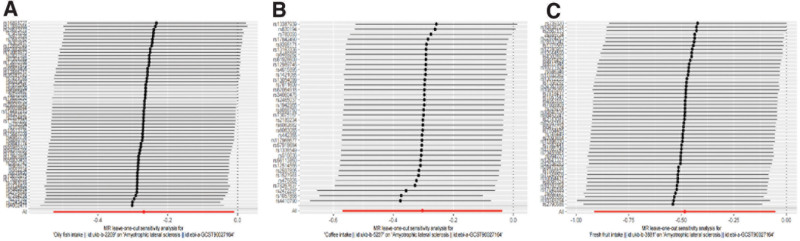
The results of leave-one-out analyses (A) Oil fish intake (B) Coffee intake (C) Fresh fruit intake.

Additionally, this study also found that Processed meat intake (OR:1.0159; 95% CI: 0.6696–1.5413; *P* = .9408; no outliers), Poultry intake (OR: 1.4665; 95% CI: 0.5386–3.9927; *P* = .4537; no outliers), Beef intake (OR: 1.4665; 95% CI: 0.5386–3.9927; *P* = .4537; no outliers), Non-oily fish intake (OR: 1.7370; 95% CI: 0.8202–3.6787; *P* = .1492; no outliers), Pork intake (OR: 0.9453; 95% CI: 0.7360–1.2142; *P* = .6597; no outliers), Bread intake (OR: 1.1453; 95% CI: 0.7541–1.7396; *P* = .5245 Outliers excluded: OR: 1.2129; 95% CI: 0.8520–1.7268; *P* = .2842), Cereal intake (OR: 0.8270; 95% CI: 0.5792–1.1808; *P* = .2959; No outliers) Cooked vegetable intake (OR: 0.9613; 95% CI: 0.3812–2.4243; *P* = .9333 Outliers excluded: OR: 0.5473; 95% CI: 0.2666–1.1234; *P* = .1004), Tea intake (OR: 0.9453; 95% CI: 0.7360– 1.2142; *P* = .6597; No outliers), Dried fruit intake (OR: 0.9384; 95% CI:0.6056–1.4542; *P* = .7762 Outliers excluded: OR: 0.9583; 95% CI: 0.6484–1.4165; *P* = .8309), Salad/raw vegetable intake (OR: 1.4715; 95% CI: 0.8085–2.6779; *P* = .2061; No outliers) were not associated with ALS. Table 2 displayed more detailed results of the MR analysis. In the Leave-one-out analysis, we verified the robustness of the results (Fig. 4). Among these negative results, we found the presence of heterogeneity when analyzing the causal relationship between cereal intake exposure factors and ALS, but we consider the results to be plausible as the egger intercept method indicated that there was no directional pleiotropy. Furthermore, in the analysis of heterogeneous results, we employed the random-effects model Inverse Variance Weighted (IVW) method to analyze the data. By using the random-effects model, we accounted for potential variability across studies and obtained robust estimates of the causal effects. This approach ensures the reliability and generalizability of our findings, considering and addressing the presence of heterogeneity in the results. As shown in Table 2, the results of the MR-PRESSO analysis were greatly consistent with the results of the IVW model.

**Table 2:** The results of Mendelian randomization analyses.

ID	Exposure	Used SNPs	Inverse variance weighted method		Weighted median method		MR-Egger method		Cochrane’s Q test		Pleiotropy			MR-PRESSO a							Outliers excluded b										
			OR (95% CI)	*P* value	OR (95% CI)	*P* value	OR (95% CI)	*P* value	Q	*P* value	MR-Egger intercept	SE	*P* value	Raw			Outliers	Outlier-corrected			Inverse variance weighted method		Weighted median method		MR-Egger method		Cochrane’s Q test		Pleiotropy		
														Casual estimate	SD	*P* value		Casual estimate	SD	*P* value	OR (95% CI)	*P* value	OR (95% CI)	*P* value	OR (95% CI)	*P* value	Q	*P* value	MR-Egger intercept	SE	*P* value
ukb-b-6324	Processed meat intake	23	1.0159 (0.6696–1.5413)	.9408	4.6896 (1.0627–1.7848)	.8182	4.6897 (0.5829–37.7282)	.1611	29.6061	.1284	−0.0232	0.0158	.1575	0.0158	0.2127	.9414	NA	NA	NA	NA	NA	NA	NA	NA	NA	NA	NA	NA	NA	NA	NA
ukb-b-8006	Poultry intake	7	1.4665 (0.5386–3.9927)	.4537	0.9468 (0.3006–2.9820)	.9256	1070501914608.13 (57.1461–2.005E + 22)	.0702	9.2779	.1585	−0.2962	0.1308	.0729	0.1289	0.4964	.8026	NA	NA	NA	NA	NA	NA	NA	NA	NA	NA	NA	NA	NA	NA	NA
ukb-b-2862	Beef intake	15	0.9233 (0.5106–1.6696)	.7918	0.7064 (0.3310–1.5077)	.3689	0.3379 (0.0127–9.0197)	.5286	18.0071	.2065	0.0127	0.0209	.5521	−0.0956	0.2891	.7451	NA	NA	NA	NA	NA	NA	NA	NA	NA	NA	NA	NA	NA	NA	NA
ukb-b-17627	Non-oily fish intake	11	1.7370 (0.8202–3.6787)	.1492	1.7131 (0.6711–4.3728)	.2602	1.6287 (0.0390–67.8865)	.8035	15.0061	.1318	0.0008	0.0232	.9732	0.5522	0.3829	.1798	NA	NA	NA	NA	NA	NA	NA	NA	NA	NA	NA	NA	NA	NA	NA
ukb-b-2209	Oily fish intake	57	0.7842 (0.5916–1.0396)	.0910	0.7594 (0.5424–1.0634)	.1091	0.3665 (0.1142–1.1763)	.0970	66.3710	.1401	0.0113	0.0086	.1930	−0.2463	0.1403	.0842	rs12983532 rs4510068 rs9606833	−0.2708	0.1287	.0397	0.7647 (0.5905–0.9904)	.0420	0.7563 (0.5384–1.0625)	.1073	0.4472 (0.1552–1.2892)	.1420	72.3928	.0693	0.0080	0.0078	.3102
ukb-b-5640	Pork intake	14	0.8838 (0.3815–2.0477)	.7733	1.1047 (0.3982–3.0645)	.8484	0.0023 (9.680E–06–0.5556)	.0508	20.6086	.0810	0.0610	0.0284	.0530	−0.1235	0.4287	.7778	NA	NA	NA	NA	NA	NA	NA	NA	NA	NA	NA	NA	NA	NA	NA
ukb-b-11348	Bread intake	24	1.1453 (0.7541–1.7396)	.5245	1.0696 (0.6393–1.7895)	.7978	1.3346 (0.2045–8.7089)	.7653	45.1466	.0157	−0.0022	0.0136	.8709	0.2001	0.2107	.3501	rs11628639 rs13016665 rs13023099 rs62091167 rs7276867 rs9662365	0.1929	0.1470	.2024	1.2129 (0.8520–1.7268)	.2842	1.1236 (0.6853–1.8423)	.6441	0.9708 (0.2269–4.1525)	.9685	16.1886	.8064	0.0033	0.0107	.7599
ukb-b-15926	Cereal intake	37	0.8270 (0.5792–1.1808)	.2959	0.8694 (0.5569–1.3572)	.5379	1.1142 (0.2314–5.3645)	.8935	51.0228	.0498	−0.0043	0.0113	.7049	−0.2185	0.1699	.2058	NA	NA	NA	NA	NA	NA	NA	NA	NA	NA	NA	NA	NA	NA	NA
ukb-b-1996	Salad/raw vegetable intake	20	1.4715 (0.8085–2.6779)	.2061	1.2398 (0.5395–2.8489)	.6126	0.4599 (0.0218–9.6890)	.6247	14.4880	.5624	0.0124	0.0163	.4574	0.4334	0.3317	.2069	NA	NA	NA	NA	NA	NA	NA	NA	NA	NA	NA	NA	NA	NA	NA
ukb-b-8089	Cooked vegetable intake	10	0.9613 (0.3811–2.4243)	.9333	0.5999 (0.2366–1.5213)	.2818	7.5040 (0.0003–221802.5666)	.7069	39.6893	.0005	−0.0211	0.0538	.7003	−0.0395	0.4720	.9344	rs12550717 rs2052063 rs2252508 rs2844672 rs4851029 rs838133	−0.6027	0.3152	.0881	0.5473 (0.2666–1.1234)	.1004	0.4681 (0.1758–1.2465)	.1287	59.3031 (0.0196–179496.3394)	.3473	6.6412	.6744	−0.0492	0.0428	.2832
ukb-b-5237	Coffee intake	36	0.7385 (0.5660–0.9637)	.0256	0.9227 (0.6236–1.3653)	.6875	1.1755 (0.6843–2.0194)	.5619	29.1810	.7445	−0.0087	0.0045	.0615	−0.2575	0.1287	.0528	NA	NA	NA	NA	NA	NA	NA	NA	NA	NA	NA	NA	NA	NA	NA
ukb-b-6066	Tea intake	41	0.9453 (0.7360–1.2142)	.6597	0.9460 (0.6791–1.3178)	.7428	0.9154 (0.5027–1.6670)	.7743	52.0258	.0643	0.0007	0.0056	.9084	−0.0745	0.1278	.5632	NA	NA	NA	NA	NA	NA	NA	NA	NA	NA	NA	NA	NA	NA	NA
ukb-b-16576	Dried fruit intake	37	0.9384 (0.6056–1.4542)	.7762	1.2928 (0.7697–2.1715)	.3317	3.7796 (0.5112–27.9437)	.2003	75.4144	.0006	−0.0172	0.0123	.1700	−0.1173	0.2175	.5926	rs11772627 rs1582322 rs7808471 rs7829800	−0.1587	0.1995	.4310	0.9046 (0.6058–1.3507)	.6240	1.0914 (0.6443–1.8488)	.7450	1.4284 (0.2108–9.6807)	.7172	50.5496	.0545	−0.0056	0.0117	.6351
ukb-b-3881	Fresh fruit intake	50	0.5947 (0.3733–0.9473)	.0287	0.9059 (0.5101–1.6086)	.7357	0.3737 (0.0666–2.0951)	.2684	80.4041	.0054	0.0044	0.0080	.5855	−0.4949	0.2286	.0349	rs1620977 rs817223	−0.4592	0.2112	.0344	0.6165 (0.4007–0.9487)	.0278	0.9457 (0.5222–1.7129)	.8540	0.7582 (0.1513–3.7998)	.7378	62.5394	.0926	−0.0019	0.0074	.7951

CI = confidence interval, NA = not available, OR = odds ratio, SNPs = single-nucleotide polymorphisms.

## 4. Discussion

The key discovery from this Mendelian Randomization analysis is oily fish intake, coffee intake, and fresh fruit intake are protective factors for ALS and have a causal relationship with ALS. Additionally, meat intake (processed meat intake, poultry intake, beef intake, pork intake and non-oily fish intake), staple food intake (bread intake and cereal intake), vegetables intake (cooked vegetable intake, salad/raw vegetable intake), fruits intake (dried fruit intake), beverage intake (tea intake) were not associated with ALS. There have been Mendelian Randomization analysis conducted on the potential risk or protective factors associated with ALS such as lipids,^[27]^ alcohol drinking,^[28]^ smoking,^[29]^ and so on. However, there are few studies involving meat intake, staple food intake, fruit intake, vegetable intake, and beverage intake.

Amyotrophic lateral sclerosis (ALS) is a progressive motor neuron disease with an insidious onset, leading to a shortened life expectancy, diminished quality of life, and potential development of psychological disorders in patients. The conclusions of our study can help clinicians to improve the health education of patients with ALS and change their eating habits (such as increasing fruit intake, oily fish intake, and appropriate coffee intake). Additionally, many genes have been identified as causative or risk factors for ALS. So, it is also significant to reduce the risk of developing ALS by adjusting dietary habits for those at high genetic risk for ALS. Hence, this investigation holds crucial implications for enhancing comprehension regarding the safeguarding aspects associated with ALS.

An Italian study has linked the risk of ALS to the frequency of meat intake, including red meat, pork, and processed meat.^[11]^ Conversely, a prospective study identified poultry intake as a protective factor for ALS.^[17]^ However, our study did not find a causal relationship between meat intake (including processed meat, beef, pork, and poultry) and ALS. This discrepancy may be attributed to the presence of multiple confounding factors in observational studies.

A study based on a case-control design found that the intake of a wide range of cereals was associated with an increased risk of ALS.^[17]^ Therefore, we analyzed the effect of cereal intake, as well as bread, a staple food commonly used in Europe, on ALS. In the MR analyses, no causal relationship was found between staple foods (bread intake and cereal intake) and ALS risk.

Several studies have indicated that increased fish consumption could potentially act as a protective factor against ALS.^[30,31]^ Omega-3 fatty acids found in fish have demonstrated anti-inflammatory effects and play a key role in maintaining cerebral functions and promoting brain development by supporting neurogenesis and neuroplasticity.^[32,33]^ Additionally, fish is a good source of protein and other nutrients that are important for overall health. Our analyses using MR also identified a causal relationship between consumption of oily fish and ALS. This suggests that including more oily fish in one’s diet may have a protective effect against this disease. Conversely, no causality was found between the non-oily fish and ALS. We attribute the discrepancies in MR studies to variations in polyunsaturated fatty acid content. Compared with non-oily fish, oily fish contains more neuroprotective Omega-3 fatty acids, thereby affecting the onset of ALS. Further research is needed to fully understand the relationship between fish consumption and ALS risk.

Consumption of fruits and vegetables can reduce the occurrence of ALS through various mechanisms. Initially, fruits and vegetables packed with antioxidants can impact the development of ALS, as there is also evidence suggesting a link between ALS and oxidative stress.^[17,29]^ An analysis of ALS patients at the outset revealed that an increased intake of antioxidants correlated with higher ALSFRS-R scores or forced vital capacity percentage.^[34]^ Furthermore, Okamoto K and colleagues discovered that a higher consumption of antioxidant-rich foods like fruits and vegetables could guard against ALS.^[14]^ Subsequently, fruits and vegetables are abundant in a range of vitamins, which have been linked to ALS in a sequence of investigations.^[35,36]^ Lastly, the consumption of fruits and vegetables enriched with carotenoids may aid in averting or forestalling the onset of ALS.^[16]^ Our research study implemented the MR analysis technique to identify a cause-and-effect relationship between fresh fruit intake and ALS, but not dried fruit intake. However, no causal relationship between vegetables intake and ALS was found.

In our study, we included two sets of beverage-related GWAS data (coffee and tea intake), because they both contain an important compound — caffeine. Caffeine has antioxidant properties,^[37,38]^ which may counteract the oxidative stress associated with aging and risk of developing neurodegenerative diseases. Besides, caffeine has a positive effect on muscle and upper and lower motor neuron excitability.^[39]^ A case-control study from Italy suggested that coffee and tea consumption may decrease the risk of ALS.^[11]^ Similarly, Beghi et al^[18]^ reported an inverse correlation between coffee intake and ALS risk. However, not all studies have shown an association between beverage intake (coffee or tea) and ALS.^[40–42]^ In our MR study, we found a causal relationship between coffee intake and ALS, but not tea intake. We believe that the reason for this result may be due to the caffeine content. There may be a certain threshold of caffeine concentration.

Furthermore, it is crucial to underscore that apart from antioxidants, lipids, caffeine, and vitamins, the intestinal microflora is also an important pathway for dietary factors to affect the prognosis of ALS patient.^[43,44]^ Wu et al^[45]^ conducted a significant investigation showcasing the association between ALS and the gut microbiome. In the SOD1 mouse model, they made an observation of impaired functionality in the intestinal barrier, along with a decline in the abundance of bacteria responsible for producing butyrate. Given that butyrate governs energy metabolism and immune functions, it could potentially contribute to neurological disorders.^[45]^

MR analyses can provide more reliable evidence of causality than observational studies. This is largely because genetic variants associated with risk factors are randomly assigned at birth and thus have a lifelong impact on the individual. As a result, MR analyses are less vulnerable to confounding factors, thereby enhancing the reliability and validity of the findings.^[20]^ Therefore, this approach can help identify potential targets for intervention and inform public health strategies aimed at preventing or managing these conditions. By utilizing IVs in this manner, researchers can improve the rigor and reliability of their findings, ultimately leading to more effective interventions and policies for improving public health. However, it is essential to accurately grasp the connection between MR and randomized controlled trials. MR serves as a valuable addition to randomized controlled trials, rather than a replacement. Thus, we should approach this finding with care.

The research in question is subject to some limitations. Initially, the participants involved in this study were all of European origin, which constrains the application of the results to diverse ethnic communities. Additionally, it is crucial to acknowledge the restrictions associated with categorizing the diverse forms of dietary intake and discerning the collective impacts of various dietary components. Lastly, while MR techniques can determine causality, they are unable to elucidate the underlying mechanisms through which dietary factors influence ALS. Additional research is necessary to explore the mechanisms that contribute to the impact of various dietary factors on the progression of ALS.

## 5. Conclusion

This research found that oily fish intake, fresh fruit intake and coffee intake were linked to a decreased risk of ALS. Conversely, meat intake (processed meat intake, poultry intake, beef intake, pork intake, non-oily fish intake and oily fish intake), staple food intake (bread intake and cereal intake), vegetables intake (cooked vegetable intake, salad/raw vegetable intake), fruits intake (dried fruit intake), beverage intake (tea intake) showed no significant association with ALS.

## Author contributions

**Conceptualization:** Zhaoyi Jing.

**Data curation:** Zhaoyi Jing.

**Formal analysis:** Zhaoyi Jing.

**Funding acquisition:** Zhaoyi Jing.

**Investigation:** Zhaoyi Jing, Jing Teng.

**Methodology:** Zhaoyi Jing.

**Project administration:** Zhaoyi Jing.

**Resources:** Zhaoyi Jing.

**Software:** Zhaoyi Jing.

**Supervision:** Xianghua Qi, Jing Teng.

**Validation:** Zhaoyi Jing.

**Visualization:** Zhaoyi Jing.

**Writing – original draft:** Zhaoyi Jing.

**Writing – review & editing:** Xianghua Qi, Jing Teng.
